# Associations of Sleep Quality and Frailty among the Older Adults with Chronic Disease in China: The Mediation Effect of Psychological Distress

**DOI:** 10.3390/ijerph17145240

**Published:** 2020-07-20

**Authors:** Peipei Fu, Chengchao Zhou, Qingyue Meng

**Affiliations:** 1 School of Health Care Management, Cheeloo College of Medicine, Shandong University, NHC Key Laboratory of Health Economics and Policy Research, Jinan 250012, China; fupeipei@sdu.edu.cn; 2 School of Public Health, Cheeloo College of Medicine, NHC Key Lab of Health Economics and Policy Research, Shandong University, Jinan 250012, China; 3 China Center for Health Development Studies, Peking University, Beijing 100191, China; qmeng@bjmu.edu.cn

**Keywords:** frailty, sleep quality, psychological distress, mediation effect

## Abstract

Frailty affects the elderly and leads to adverse health outcomes. Preliminary evaluations have suggested that sleep quality and psychological distress are predictors of frailty among older adults. However, the mechanisms by which sleep quality affect frailty had not been fully addressed in the previous research. This study aimed to explore the mediation effect of psychological distress on the association between sleep quality and frailty among the elderly with chronic diseases in rural China. A total of 2346 old adults were included in the analysis. Frailty status was measured by Fried Phenotype criteria. Sleep quality was assessed by Pittsburgh Sleep Quality Index (PSQI), and psychological distress was examined by Kessler Psychological Distress Scale (K10). Ordinal logistic regressions were performed to assess the relationships between sleep quality and frailty. Mediation test was also conducted by bootstrap method. The prevalence rate of frailty among the elderly with chronic diseases was 21% in rural China. Compared with the elder of robust status, respondents identified as having frailty have lower SES, less vigorous physical activity, and worse self-reported health status. Poor sleep quality was a significant predictor of frailty with mediators (OR = 1.44, 95% CI = 1.19–1.76). Mediation analysis suggested that psychological distress mediated 41.81% of total effect between sleep quality and frailty. This study indicated that poor sleep quality was significantly related to frailty, and psychological was a mediator of this association. However, we could not investigate causal relationships between variables since this was one cross-sectional study. These findings suggested that an early detection of sleep problems and also psychological disorders should be taken to prevent frailty among the rural older adults in China.

## 1. Introduction

Frailty in older adults is one complicated condition accompanied with aging and characterized with progressive decline in physiological reserves and resistance to stressors, which are related to increased morbidity and mortality [1,2]. Numerous evidences have linked frailty to negative health, such as disability, hospitalization, higher mortality rates, and threaten quality of life of older adults [3,4]. Previous studies indicate that chronic diseases are positively associated with the onset of frailty [5,6,7]. A study in Singapore demonstrate that frailty prevalence in older adults with diabetes is twice of that in general population [8]. In addition, frail individuals with chronic disease are demonstrated to have higher risk of mortality [9]. Thus, frailty among the individuals with chronic disease requires more attention. Moreover, studies suggest frailty is reversible and can be treated and delayed by interventions, such as health promotion, nutrition, physical, and social support [10,11,12,13]. Therefore, with the rapid growth of aging population, special attentions should be paid on exploring risk factors and preventing frailty among the older adults with chronic diseases.

Previous studies have explored risk factors associated with frailty. A systematic review suggests that there is a positive association between sleep quality and frailty [14]. On study reports, long sleep durance (≥10 h) is associated with higher risk of pre-frail and frail status [15]. In China, around 37.75% of the older adults living in urban areas are suffering from poor sleep quality, such as sleep disorder, sleep disturbance, and insomnia [16,17]. A cross-sectional study performed in urban China report similar results that poor sleep quality and long sleep duration increased the prevalence of pre-frailty and frailty [18]. Given the concerning relationship between poor sleep quality and frailty, questions still remain unanswered regarding the underlying pathways between sleep quality and frailty among older adults in rural China. 

Findings from empirical studies suggest one potential mediating role of psychological distress. Previous studies in rural China suggest that abnormal sleep is associated with anxiety and depressive symptoms [19,20]. In particular, sleep duration is one predictor of anxiety, and insufficient sleep is a causal factor in depression [21,22]. Additionally, insomnia will increase risks of mental illness [23,24]. It has also been demonstrated that certain forms of psychological disorder including depression and suicidal ideation are associated with an increased risk of frailty [25,26]. Therefore, we hypothesize that psychological distress may mediate the association of sleep quality and frailty among the elderly with chronic diseases. 

To the best of our knowledge, literatures on the underlying mechanisms to explain how sleep quality is associated with frailty are limited and should be further examined, in particular with mediating effects. A recent research demonstrated that mood regulation on depression and anxiety is a potential mediator of physical activity on sleep quality [27]. Furthermore, the frail older adults with chronic diseases living in rural areas deserve more attention since they have a lack of health knowledge and access to health-care management, which makes them more vulnerable. The objectives of this study are: (1) to assess the association between sleep quality and frailty among older adults with chronic diseases in rural China and (2) to explore the mediation effect of psychological distress between sleep quality and frailty. 

## 2. Materials and Methods

### 2.1. Study Design and Sample

Data were drawn from a cross-sectional study conducted in Shandong Province, the second largest province in China. The survey was conducted in 2019 as the first wave of the Longevity and Aging Cohort Study, which aims to investigate health status of the elderly living in rural areas of China. Multistage random sampling was used to recruit senior citizens above 60 years old and living at rural areas. To do so, three cities were first chosen representing different economic development levels and location within the province. Within each city, five townships were randomly chosen and four villages in each township were finally chosen as our study sites. Face-to-face interview were conducted among respondents, and then a brief physical examination was done by our training investigators. 

The criteria for inclusion in this study were as follows: (1) aged 60 years or above; (2) older individual with at least one chronic disease including hypertension, diabetes, dementia, waist and leg pain, chronic gastritis, stroke, and cancer. To obtain accurate data, we exclude respondents who could not answer the questionnaire independently. All of the completed questionnaires were carefully checked by the supervisors after the interview each day. In total, 3600 individuals were recruited and 3243 completed the whole survey, with a response rate of 90.05%. Of the respondents, 896 respondents were excluded for without chronic conditions and one respondent was excluded from our analysis due to the lack of data in psychological distress survey. Finally, 2346 respondents were included in the final statistical analysis. 

### 2.2. Frailty Status Measurements

Frailty status was determined based on the Fried Phenotype criteria which assess frailty in five aspects: unintentional weight loss, exhaustion, physical activity, walk time, and grip strength [28,29]. Frailty was identified as one meet three and more criterions, pre-frailty was determined as individual meet one and two of the criteria, and robust was defined as one meet none of the characteristics. In this study, five criterions were measured as follows (see Appendix A Table A1 for cut-off points):

(1) Unintentional weight loss. This criterion was defined as the unintentional weight loss more than 5% over last year by asking questions: “what was your weight last year” and “Did you lose weight intentionally?” 

(2) Exhaustion. Defined as self-reported “feel exhausted to do everything” or “I could not go on with my life” more than 3 times a week will receive a score of 1. 

(3) Weakness. Defined as grip strength smaller than the lowest 20% by gender and Body Mass Index (BMI). The grip strength was assessed by dynamometer for three times and took average of the measurements.

(4) Slowness. Slowness was defined by the speed of walking 4.6m adjusting gender and height.

(5) Low physical activity. Defined according to International Physical Activity Questionnaire-short version [30,31]. Each activity was assigned a metabolic equivalent (MET) energetic cost value. The total Kcals of activity for last seven days was calculated by total minutes of physical activities times MET score adjusting gender. 

### 2.3. Sleep Quality 

Sleep quality was assessed by Pittsburgh Sleep Quality Index (PSQI) [32]. This scale consisted 19 self-reported questions in seven dimensions including sleep duration, time to fall asleep, sleep disturbance, sleep efficiency, daytime dysfunction, medications to sleep, etc. [33]. The total score range is 0–20 and the higher of the PSQI score represents worse sleep quality. In this study, the cut off value was set at 7, and individual’s PSQI score greater than 7 represented poor sleep quality [34]. 

### 2.4. Psychological Distress

Psychological health status was examined by Kessler Psychological Distress Scale (K10) which was used to assess psychological distress and has been widely applied in screening psychological health for elders [35,36]. This questionnaire contains 10 items with five-point scale to measure frequency of symptoms associated with nonspecific mental health conditions, such as anxiety and stress in the previous month by asking questions: “Do you often feel nervous?”, “Do you often feel helpless?”, “Do you often fell depressed?”, “Do you often feel worthless?”, etc. The total score range is 0–50 and higher score were classified as poor psychological health status. As previous study suggested, psychological status was classified into four categories based on K10 score: no psychological distress (10–15), mild psychological distress (16–21), moderate psychological distress (22–29), and severe psychological distress (30–50) [37]. 

### 2.5. Statistic Analysis

**Figure 1:**
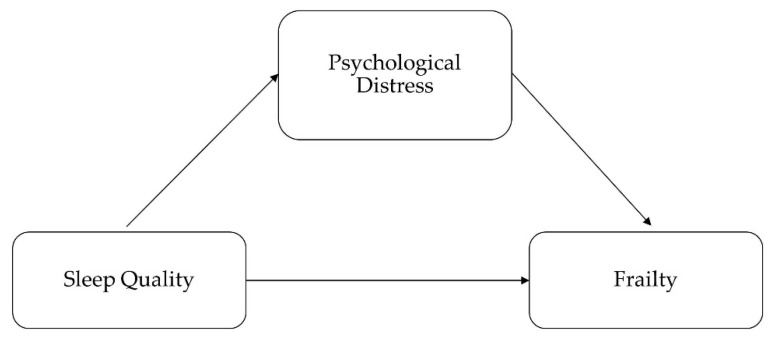
Diagram of the mediation model.

The data were analyzed by STATA 14.2 (Stata Corp; College Station, TA, USA). Descriptive statistical analysis with Chi-square test was performed to test variances of demographic characteristics by different frailty groups. Ordinal logistic regressions were used to investigate associations between frailty and sleep quality, and effects mediated by psychological distress. We performed the test proposed by Baron and Kenny [38]. Specifically, three regression models with control variables were conducted as shown in Figure 1: firstly, ordinal logistic regression was conducted to test the association between sleep quality and frailty; secondly, ordinal logistic regression was performed to exam association between psychological disorder on sleep quality; finally, ordinal logistic regression was conducted to explore relationship between sleep quality and frailty with psychological disorder included in the model. The mediation effect test model was conducted as follows:

(1) $$Y=cX+e_{1}$$

(2) $$M=aX+e_{2}$$

(3) $$Y=c^{\prime }X+bM+e_{3}$$

The mediation effects were tested by bootstrap method [39,40]. Odds ratio (OR) values with 95% confident intervals (CI) were also presented. The ordinal logistic regressions were adjusted for social demographic characteristics with gender, age, education, occupation, marital status, and household income. Variables including activity, alcohol intake, smoking, BMI index, and self-reported health status were also regarded as covariates in the statistical analysis. In addition, we conducted a sensitivity analysis on psychological distress and sleep quality, and on sleep quality and frailty with psychological distress as a mediator using categorical and continuous K10 scores, respectively.

### 2.6. Ethical Consideration

This study protocol was approved by the Ethical Committee of Shandong University School of Public Health (No. 20,181,228). All respondents were fully informed with written consent for participation prior to the face-to-face interview.

## 3. Results

The socio-demographic characteristics of the respondents with Chi-square test results were presented in Table 1. The percentage of respondents identified as robust, pre-frailty, and frailty were 15%, 64%, and 21%, respectively. About 65% of the participants were female, and average age of frailty elders was higher than elders without frailty. Nearly half of the participants had not attended school. More than half of the participants with poor self-reported health status suffered from frailty. There was no significant difference in BMI (*p* = 0.671) and smoking habits (*p* = 0.506) among frail older adults. Furthermore, significant differences existed among respondents with different levels of household income, physical activity, and self-reported health status.

### 3.1. Effects of Sleep Quality on Psychological Distress

Table 2 reported the estimation results of three models to test the mediating effect of psychological distress on the association between sleep quality and frailty. Specifically, model 2 presented odds ratios of the associations between sleep quality and psychological distress. The estimation results in model 2 suggested that compared with those with high sleep quality (PSQI < 7), older adults with poor sleep quality (PSQI ≥ 7) were more likely to be of psychological distress (OR = 3.48, CI = 2.89, 4.18, *p*-value < 0.001), after controlling for the potential confounding variables. 

### 3.2. Effects of Sleep Quality and Psychological Distress on Frailty 

Model 3 tested associations between sleep quality, psychological distress, and frailty. The analysis results indicated that worse sleep quality (OR = 1.44, CI = 1.19, 1.76, *p*-value < 0.001) was significantly associated with frailty development (robust, pre-frailty, and frailty). Similar relationship was observed from psychological distress on frail older adults with chronic diseases, as OR = 1.63, CI = 1.29, 2.05, *p*-value < 0.001 for mild, OR = 2.34, CI = 1.81, 3.02, *p*-value < 0.001 for moderate, and OR = 4.43, CI = 3.15, 6.22, *p*-value < 0.001 for severe. Model 1 examined relationships between sleep quality and frailty without psychological distress; the result indicated that the odds of having pre-frailty and frailty will increase by a factor of 1.84 for individuals who had poor sleep quality (OR = 1.84, CI = 1.53, 2.23, *p*-value < 0.001). Additionally, the sensitivity analysis including the categorical and continuous K10 scores reported a similar association between sleep quality and psychological distress, and between sleep and frailty (Tables S1 and S2).

### 3.3. Mediation Effects of Psychological Distress on Frailty

The mediation effects by psychological distress were tested by bootstrap, and results indicated psychological distress partially mediated 41.81% of the total effects of sleep quality on frailty. The odds ratio of PSQI in Table 2 decreased from 1.84 (CI = 1.53, 2.23) to 1.44 (CI = 1.19, 1.76) as psychological distress included in the full model. This indicated that the effect of sleep quality on frailty was partially mediated by psychological distress. Participants who had poor sleep quality and mild to severe psychological distress had a more sever frailty. All three statistic regression models were adjusted for socio-demographic variables, health behavior, and health status variables.

## 4. Discussion

In this cross-sectional study, after adjustment for a variety of confounders, we have identified that both sleep quality and psychological distress were significantly associated with frailty status. Our study also suggested that sleep quality was highly associated with psychological distress. Therefore, we revealed that psychological distress mediated partially of the relationship between sleep quality and frailty. Furthermore, our study found the prevalence rate of frailty in the elderly with chronic diseases (21%) was higher than among general Chinese elders (18%) [41]. Additionally, the present study suggested that older female had a higher prevalence of frailty (19.22%) than that of male (15.74%), which has been widely found in previous studies [19,42]. One meta-analysis indicated that the pooled prevalence of frailty was 8% in males and 11% in females [43]. Therefore, the frail elders with chronic diseases should be addressed for policy makers.

Consistent with previous research, our study also found that poor sleep quality was associated with frailty [44,45]. We found that 75.6% of older adults who had poor sleep quality were accompanied with pre-frail and frail status. Previous studies also indicated sex difference existed in the relationship between poor sleep quality and frailty; specifically, women were associated with higher risk of frailty than men [46,47]. Similarly, this study revealed that the prevalence of poor sleep quality of women was higher than men, but sex difference was not significant between sleep quality and frailty. 

In line with previous research, our results showed that sleep quality was associated with psychological distress and individuals with poor sleep quality were significantly related to severe psychological distress [48]. Our study recognized that the prevalence rate of poor sleep was significantly higher in individuals with psychological distress (80.4%) than that without psychological distress (19.6%) among older adults. This extended previous finding that sleep quality negatively correlated with individual’s mood, since sleep problems threaten quality of life and associated with mood in older adults [49]. Our results could be explained by the fact that poor sleep quality, such as trouble in falling asleep and short sleep duration, will impair mood and increase anxiety with daytime fatigue, therefore causing psychological disorders [50,51]. 

One potential mechanism was psychological disorder explained how sleep quality impact frailty was through inadequate activity. Previous literatures suggested that poor sleep quality would result in depression and decreased physical activity, increasing the risk of negative health conditions [52,53]. In particular, a longitudinal study suggested that long sleepers were characterized with inadequate activity and muscle strength, and slow walking speed which contributing to frailty [54]. Such findings were supported in our study as participants engage in vigorous activity associated with lower frailty.

Another mechanism that poor sleep quality lead to frailty among older adults with chronic diseases was explained by oxidative stress. Literature had proved that oxidative stress was a predictor of age-related diseases including cardiovascular diseases, Alzheimer’s diseases, and other neurodegenerative disorders [55], and lead to musculoskeletal damage which in turn results in frailty [56]. In addition, oxidative stress contributed to sleep disorder and sleep disorder with depression were confounding determinants of fatigue [57]. Ultimately, senior residents with pre-frail and frail status were more likely to suffer from chronic comorbidities, which were triggered by oxidative stress [58].

Based on the results and interpretation, we have verified the hypothesis that poor sleep quality contributes to frailty and partially mediated by psychological distress in rural China. Therefore, we recommend health policies aiming to prevent elders from falling into frailty and enhance healthy aging. First, joint policy avocation should be addressed to strengthen the early diagnosis and treatment of rural elders with sleep problems and psychological disorders. Moreover, healthy aging could be enhanced by promoting healthy behaviors including participating in moderate to vigorous physical exercise regularly to improve physical capacity and prevent frailty in elders. Accordingly, policies should be initiated on the development and maintaining of healthy behaviors, especially encouraging engagement in regular physical activities to reduce chronic diseases and improve sleep quality. Additionally, psychological health of the elderly in rural China should be highlighted through strategic policy interventions such as social support from family and community. Public environment also needs to be improved to upgrade overall living conditions for rural residents. 

Our study has several limitations. We could not investigate causal relationships between variables since this was one cross-sectional study. Due to resource constraints, this study was conducted in a single province, which may limit the generalizability of the study results to other areas in China. In addition, variables values collected such as health status and recall of sleep habits in previous month were self-reported; therefore, bias might exist in the values. Future research should be conducted to explore other underling mechanisms to explain how sleep quality affect frailty among older adults with chronic diseases so as to provide evidence for policy interventions. 

## 5. Conclusions

This study was the first to explore mediation effect of psychological distress on associations between sleep quality and frailty among the elderly with chronic diseases in rural China. We found poor sleep quality was significantly associated with frailty, and psychological distress was a mediator of this association. These findings implied that an early detection of sleep problems and also psychological disorders by regular monitoring and timely interventions, such as improving social support from community or treatment would be helpful for preventing frailty among the rural older adults in China.

## Figures and Tables

**Table 1:** Demographic characteristics of older people by category of frailty status in rural Shandong, China (*n* = 2346).

Characteristics	All *N* (%)	Robust	Pre-Frailty	Frailty	*p*-Value
		*n* (%)	*n* (%)	*n* (%)	
Total	2346 (100)	342 (15)	1504 (64)	500 (21)	
Gender					0.090
Male	820 (35)	109 (32)	550 (37)	161 (32)	
Female	1526 (65)	233 (68)	954 (63)	339 (68)	
Age (years)					<0.001
Mean ± SD	70.15 (6.05)	68.19 (5.03)	70.08 (6.00)	71.70 (6.41)	
Education					<0.001
Illiteracy	963 (41)	117 (34)	627 (42)	219 (44)	
Junior school	948 (40)	136 (40)	600 (40)	212 (42)	
Senior school+	435 (19)	89 (26)	277 (18)	69 (14)	
Occupation					<0.001
Unemployed	1026 (44)	99 (29)	632 (42)	295 (59)	
Farmers	1320 (56)	243 (71)	872 (58)	205 (41)	
Marital Status					0.209
Married	1737 (74)	266 (78)	1108 (74)	363 (73)	
Other	609 (26)	76 (22)	396 (26)	137 (27)	
Household income/year					<0.001
<7 K	787 (33)	65 (19)	513 (34)	209 (42)	
7–15 K	657 (28)	114 (33)	404 (27)	139 (28)	
>15 K	902 (39)	163 (48)	587 (39)	152 (30)	
Activity					<0.001
Moderate	1166 (49)	134 (39)	697 (46)	335(67)	
Vigorous	1180 (51)	208(61)	807 (54)	165 (33)	
Drinking					<0.001
None	1708 (72)	247 (72)	1067 (71)	394 (79)	
Ever	178 (8)	24 (7)	122 (8)	32 (6)	
Current	460 (20)	71 (21)	315 (21)	74 (15)	
Smoking					0.506
None	1652 (70)	249 (73)	1048 (70)	355(71)	
Ever	262 (11)	41 (12)	170 (11)	51 (10)	
Current	432 (19)	52 (15)	286 (19)	94 (19)	
BMI					0.671
Mean ± SD	24.72 (4.05)	24.62 (3.46)	24.78 (4.08)	24.63 (4.31)	
Self-reported Health Status					<0.001
Poor	786 (33)	53 (15)	455 (30)	278 (56)	
Medium	740 (32)	109 (32)	499 (33)	132 (26)	
Good	820 (35)	180 (53)	550 (37)	90 (18)	

Notes: Senior school+: represents senior school and above.

**Table 2:** The mediating effect of psychological distress on the association between sleep quality and frailty among elders with chronic diseases (*n* = 2346).

Variables	Model 1		Model 2		Model 3	
	Frailty		Psychological Distress		Frailty	
	OR	95% CI	OR	95% CI	OR	95% CI
Sleep quality	-	-	-	-	-	-
PSQI < 7 (ref.)	-	-	-	-	-	-
PSQI ≥ 7	1.84 ***	(1.53, 2.23)	3.48 ***	(2.89, 4.18)	1.44 ***	(1.19, 1.76)
Psychological distress		-	-	-	-	-
No (ref.)	-	-	-	-	-	-
Mild	-	-	-	-	1.63 ***	(1.29, 2.05)
Moderate				-	2.34 ***	(1.81, 3.02)
Severe	-	-	-	-	4.43 ***	(3.15, 6.22)

Notes: OR represents the odds ratio, and 95% CI represents 95% confidence intervals. The above models adjusted for socio-demographic variables, drinking, smoking, BMI, chronic diseases, and self-reported health status variables. *** *p* < 0.001.

## Supplementary Materials

The following are available online at https://www.mdpi.com/1660-4601/17/14/5240/s1, Table S1: Model estimation using categorical K10 vs. continuous K10, Table S2: Model estimation using categorical K10 vs. continuous K10.

## Appendix A

**Table A1:** Cut-off points for frailty measurements.

Measures	Cut-Off Points for Frailty	
Weakness	Male	
	Grip Strength (Kg)	BMI
	<29	≤24
	<30	24.1–26
	<30	26.1–28
	<32	>28
	Female	
	Grip Strength (Kg)	BMI
	<17	≤23
	<17.3	23.1–26
	<18	26.1–29
	<21	>29
Slowness	Male	
	Time to walk 4.6 m (seconds)	Height (cm)
	≥7	≤173
	≥6	>173
	Female	
	Time to walk 4.6 m (seconds)	Height (cm)
	≥7	≤159
	≥6	>159
Low physical Activity	Male	
	Total Kcals (per week)	-
	<383	-
	Female	
	Total Kcals (per week)	-
	<270	-
