# Supplementary material for: Associations of Sleep Quality and Frailty among the Older Adults with Chronic Disease in China: The Mediation Effect of Psychological Distress
Source: Int J Environ Res Public Health. 2020 Jul 20;17(14):5240. doi: 10.3390/ijerph17145240 (PMC7400303; doi:10.3390/ijerph17145240)
Supplement: Supplementary file 1 [file ijerph-17-05240-s001.pdf]

Sensitivity Analysis

Table S1 Model estimation using categorical K10 vs continuous K10

Predictors	Coeff	p-value	95% CI
Sleep quality			
PSQI<7 (ref.)			
PSQI≥7	1.27	<0.001	1.11-1.42
Estimation on continuous K10	Coeff		
Sleep quality			
PSQI<7 (ref.)			
PSQI≥7	0.53	<0.001	0.47-0.60

Table S2 Model estimation using categorical K10 vs continuous K10

Predictors	Model with mediators			Model without mediators		
	OR	p-value	95% CI	OR	p-value	95% CI
Sleep quality						
PSQI<7 (ref.)						
PSQI≥7	1.44	<0.001	1.19-1.76	1.84	<0.001	1.53-2.23
Psychological distress						
No (ref.)						
Mild	1.63	<0.001	1.29-2.05			
Moderate	2.34	<0.001	1.81-3.02			
Severe	4.43	<0.001	3.15-6.22			
Estimation with continuous K10						
Sleep quality						
PSQI<7 (ref.)						
PSQI≥7	1.37	<0.001	1.16-1.62	1.84	<0.001	1.53-2.23
Psychological distress	1.07	<0.001	1.06-1.09			
